# Engineering drought-tolerant apple by knocking down six *GH3* genes and potential application of transgenic apple as a rootstock

**DOI:** 10.1093/hr/uhac122

**Published:** 2022-05-26

**Authors:** Lijuan Jiang, Wenyun Shen, Chen Liu, Muhammad Mobeen Tahir, Xuewei Li, Shuangxi Zhou, Fengwang Ma, Qingmei Guan

**Affiliations:** State Key Laboratory of Crop Stress Biology for Arid Areas/Shaanxi Key Laboratory of Apple, College of Horticulture, Northwest A&F University, Yangling, Shaanxi 712100, China; State Key Laboratory of Crop Stress Biology for Arid Areas/Shaanxi Key Laboratory of Apple, College of Horticulture, Northwest A&F University, Yangling, Shaanxi 712100, China; State Key Laboratory of Crop Stress Biology for Arid Areas/Shaanxi Key Laboratory of Apple, College of Horticulture, Northwest A&F University, Yangling, Shaanxi 712100, China; State Key Laboratory of Crop Stress Biology for Arid Areas/Shaanxi Key Laboratory of Apple, College of Horticulture, Northwest A&F University, Yangling, Shaanxi 712100, China; State Key Laboratory of Crop Stress Biology for Arid Areas/Shaanxi Key Laboratory of Apple, College of Horticulture, Northwest A&F University, Yangling, Shaanxi 712100, China; The New Zealand Institute for Plant and Food Research Ltd, Hawke’s Bay 4130, New Zealand; State Key Laboratory of Crop Stress Biology for Arid Areas/Shaanxi Key Laboratory of Apple, College of Horticulture, Northwest A&F University, Yangling, Shaanxi 712100, China; State Key Laboratory of Crop Stress Biology for Arid Areas/Shaanxi Key Laboratory of Apple, College of Horticulture, Northwest A&F University, Yangling, Shaanxi 712100, China

## Abstract

Drought poses a major threat to apple fruit production and quality. Because of the apple’s long juvenile phase, developing varieties with improved drought tolerance using biotechnology approaches is needed. Here, we used the RNAi approach to knock down six *GH3* genes in the apple. Under prolonged drought stress, the *MdGH3* RNAi plants performed better than wild-type plants and had stronger root systems, higher root-to-shoot ratio, greater hydraulic conductivity, increased photosynthetic capacity, and increased water use efficiency. Moreover, *MdGH3* RNAi plants promoted the drought tolerance of the scion when they were used as rootstock, compared with wild-type and M9-T337 rootstocks. Scions grafted onto *MdGH3* RNAi plants showed increased plant height, stem diameter, photosynthetic capacity, specific leaf weight, and water use efficiency. The use of *MdGH3* RNAi plants as rootstocks can also increase the C/N ratio of the scion and achieve the same effect as the M9-T337 rootstock in promoting the flowering and fruiting of the scion. Notably, using *MdGH3* RNAi plants as rootstocks did not reduce fruit weight and scion quality compared with using M9-T337 rootstock. Our research provides candidate genes and demonstrates a general approach that could be used to improve the drought tolerance of fruit trees without sacrificing the yield and quality of scion fruits.

## Introduction

Genetic improvements in the breeding of perennial woody plants are challenging to achieve because of their long growth period. New technologies (e.g. biotechnology) provide strategies to accelerate the breeding of woody plants and obtain new varieties with ideal characteristics [1–4]. Transgenic technology has been increasingly applied to agriculture. A typical example is the use of genetically modified soybeans [5]. Hence, it is effective and feasible to obtain new woody varieties using transgenic technology. In the field of fruit tree breeding, the use of genetically modified technology can circumvent the problem of interspecies hybridization, promote the improvement of fruit tree traits, and thus expand the scope of breeding by providing new avenues for fruit tree breeding.

During their growth and development, plants are often affected by various types of environmental stress, such as drought, ultraviolet radiation, high salinity, and temperature extremes. Drought, in particular, has a major effect on the growth and development of plants and impedes the development of good-quality and high-yield crops [6–8]. Plants adapt to the natural environment in regions where they have grown for long periods, and they have developed a set of physiological and ecological characteristics that are most suitable for their own growth and development, including a series of mechanisms to respond to drought. Plants can resist or endure the damage caused by drought stress in various ways [9–12], as drought can decrease the photosynthetic rate of plants, destroy the structure of chloroplasts, and restrict electron transfer and enzyme activity. Thus, photosynthetic capacity can reflect the degree of damage induced by drought stress in plants and can reflect drought tolerance [13].

Many plants adjust root structure in response to drought stress [14]. Changes in root system architecture improve the ability of plants to absorb water and enhance the drought resistance of plants under water deficits [15–17]. Generally, drought-resistant plants have roots and shoots with higher water-conducting capacity [18]. Root or shoot hydraulic conductivity can reflect the drought tolerance of plants under water-limited conditions [18, 19]. In addition, water use efficiency (WUE) is an important index of plant adaptation to drought stress [7, 20, 21]. Leaf carbon isotopic composition (δ^13^C) is widely used to quantify WUE. δ^13^C has been shown to be positively correlated with WUE in many species [7, 20, 22, 23], including apple [20]. Consequently, many studies have indicated that δ^13^C is an appropriate parameter for appraising the WUE of whole plants.

Grafting is an asexual plant reproduction technique that is widely used in horticultural plants. The most common method of grafting is to connect the vegetative organs of a plant with excellent characteristics to the stem of another rooted plant so that the two can heal and grow to form a new independent plant. The shoot piece is known as the ‘scion’ and the root piece is called the ‘rootstock’ (stock) [24–27]. Grafting has been widely applied in horticulture plants to obtain high-yield and good-quality fruits and vegetables [26–29], as it has been shown to not only improve yield and fruit quality but also increase the WUE of plants [29–31]. In addition, grafting has been shown to increase the resistance and adaptability of plants to biotic and abiotic stresses, including resistance to diseases, low and high temperatures, salinity, and drought [24, 31–38]. In fruit trees, grafting is widely used to improve stress tolerance, accelerate flowering, and shorten the juvenile phase [24]. Sugar is an energy substance that regulates plant growth and flowering transition in grape and citrus [39–41]. Sugars play an important role in plant flowering [42]. Besides, the high C/N ratio is also an important index to judge early flowering in fruit trees [42].

GH3 family proteins function in the conjugation of indole-3-acetic (IAA) to amino acids [43, 44]. The reaction of IAA conjugates with leucine, alanine, and phenylalanine is a reversible process, forming storage compounds that can be hydrolyzed to free IAA, while conjugates of IAA with glutamic or aspartate acid undergo oxidative metabolism without hydrolyzing to free IAA [43, 45–47]. In addition, GH3 family proteins work together with auxin oxidase and the conjugating enzyme Dioxygenase for Auxin Oxidation 1 (DAO1) to regulate IAA homeostasis during plant growth and development of *Arabidopsis* and rice [43, 48–50]. *DFL1*, an auxin-responsive *GH3* gene in *Arabidopsis*, has been shown to promote the light response of hypocotyls and inhibit shoot cell elongation and lateral root formation [51]. Besides their roles in growth and development, *GH3* family genes also play crucial roles in plant resistance to stresses [14]. For example, overexpression of *OsGH3.2* in rice decreases drought tolerance [52]. *OsGH3.13* was induced by drought, and *OsGH3.13*-overexpressing rice showed enhanced drought tolerance [53]. In cotton, virus-induced gene silencing (VIGS) of *GH3.5* reduces drought and salt tolerance [54].

Apple trees have one of the longest cultivation histories and are one of the most widely planted fruit trees in the world. In the Northwest Loess Plateau, drought has seriously affected the growth and production of apples [6–8]. To reduce the losses caused by drought, rootstocks with drought tolerance need to be developed. The breeding of apple rootstocks with improved drought tolerance can be achieved using biotechnology approaches. Previously, we found that knockdown of six *GH3* family genes (*MdGH3.6* and its close paralogs) from *Malus* × *domestica* increased the apple wax content and drought tolerance under short-term drought conditions [55]. In this study, we demonstrate that the knockdown of six *MdGH3* genes increases the adaptability of apple trees to prolonged drought and that the use of *MdGH3* RNAi plants as rootstocks improves scion performance under drought, compared with non-transgenic plants (GL-3) and M9-T337 rootstocks. M9-T337 is the most widely used apple dwarfing rootstock, which has contributed to the early flowering and fruit setting of the scions. However, it is sensitive to water deficits due to its shallow and weak root system [56]. In addition, when compared with the M9-T337 rootstock, *MdGH3* RNAi as rootstocks did not reduce the flowering rate, fruit size, and quality of the scion. Our results provide candidate genes for improvement of the drought tolerance of fruit trees without penalty to scion fruit yield and quality.

## Results

### Knockdown of *MdGH3* genes improves apple performance under long-term drought stress

**Figure 1:**
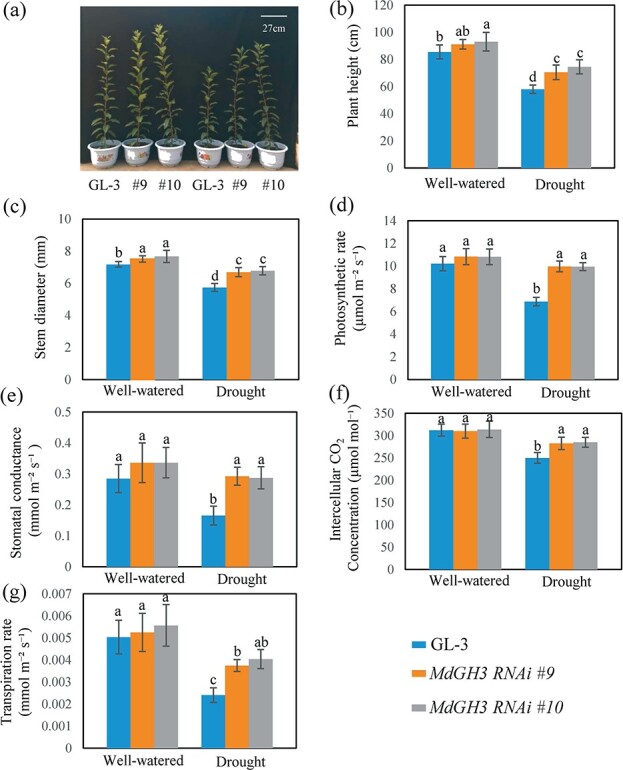
*MdGH3* RNAi plants perform better after long-term drought stress. **a** Morphology of GL-3 and *MdGH3* RNAi plants under well-watered and prolonged drought stress. **b**–**g** Plant height (**b**), stem diameter (**c**), photosynthetic rate (**d**), stomatal conductance (**e**), intercellular CO_2_ concentration (**f**), and transpiration rate (**g**) of GL-3 and *MdGH3* RNAi plants under well-watered and long-term drought stress. Plants were exposed to drought for 60 days. Error bars indicate the standard deviation (*n* = 18). One-way ANOVA (Tukey test) was performed.

To investigate whether the repression of *MdGH3* genes (*MdGH3.6* and its close paralogs) affects the tolerance of apples to long-term drought, a 2-month drought stress treatment was applied to GL-3 and *MdGH3* RNAi plants. Long-term drought significantly decreased plant height and stem diameter. Still, *MdGH3* RNAi plants were slightly taller and had greater diameters than GL-3 plants under prolonged drought (Fig. 1a–c). Under long-term drought, GL-3 plants decreased in height by 33% and in diameter by 21%. By contrast, *MdGH3* RNAi plants decreased in height by 23% and in diameter by 10% under the same drought conditions (Fig. 1b and c). Additionally, *MdGH3* RNAi plants had better photosynthetic capacity, higher stomatal conductance, higher intercellular CO_2_, and a higher transpiration rate under long-term drought stress (Fig. 1d–g). Their photosynthetic rate declined by 36% under drought, compared with a decline of 51% in GL-3 plants under drought (Fig. 1d). Under well-watered conditions, *MdGH3* RNAi plants had thicker stem diameters (Fig. 1c). However, no significant differences in plant height, photosynthetic capacity, stomatal conductance, intercellular CO_2_, and transpiration rate between GL-3 and *MdGH3* RNAi plants were observed under well-watered conditions (Fig. 1b, d–g).

**Figure 2:**
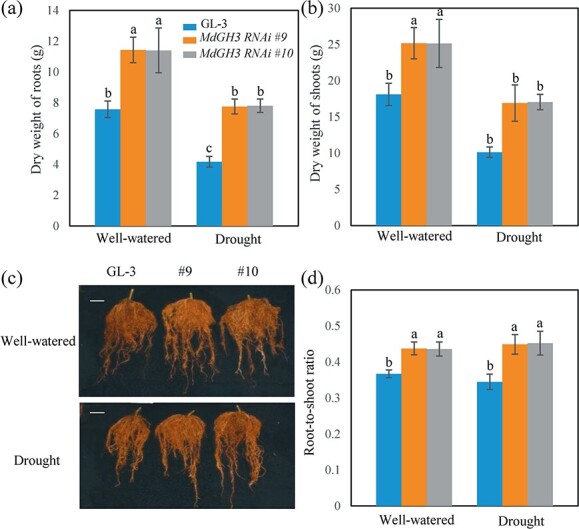
*MdGH3* RNAi plants develop a stronger root system after long-term drought stress. Dry weight of roots (**a**), dry weight of shoots (**b**), root morphology (**c**), and root-to-shoot ratio (**d**) of GL-3 and *MdGH3* RNAi plants under well-watered and long-term drought stress. Plants were exposed to drought for 60 days. Scale bars = 3 cm. Error bars indicate the standard deviation (*n* = 7). One-way ANOVA (Tukey test) was performed.

Long-term drought reduced shoot and root dry weight as well as root size (Fig. 2a–d). Specifically, the root and shoot dry weights of GL-3 plants were reduced by 45 and 44% under drought, respectively, whereas those of *MdGH3* RNAi plants were reduced by 32 and 33%, respectively (Fig. 2a and b). The root-to-shoot ratio is an indicator of plant adaptation to drought stress [18]. The root-to-shoot ratio of GL-3 plants did not differ significantly between well-watered and drought-stressed plants. However, the root-to-shoot ratio was slightly higher in drought-stressed *MdGH3* RNAi plants than in well-watered plants, suggesting that *MdGH3* RNAi plants had better performance under drought stress. More importantly, the *MdGH3* RNAi plants had a higher root-to-shoot ratio than GL-3 plants under drought stress (Fig. 2d). We also measured the ABA content of GL-3 and *MdGH3* RNAi plants under well-watered and long-term drought stress conditions (Supplementary Data Fig. S1a). We found that *MdGH3* RNAi plants contained more abscisic acid (ABA) than GL-3 plants under well-watered and long-term drought stress conditions. Previously, we found that *MdGH3* RNAi plants contain more IAA under short-term drought stress [55]. Therefore, we measured the IAA content of *MdGH3* RNAi plants under well-watered and long-term drought stress conditions. We found that *MdGH3* RNAi plants contained more IAA than GL-3 plants in leaves and roots under well-watered and long-term drought stress conditions (Supplementary Data Figs S1b and S2). Overall, these data suggest that *MdGH3* RNAi plants were more tolerant of long-term drought stress.

### Knockdown of *MdGH3* genes increases hydraulic conductivity and water use efficiency under long-term drought

**Figure 3:**
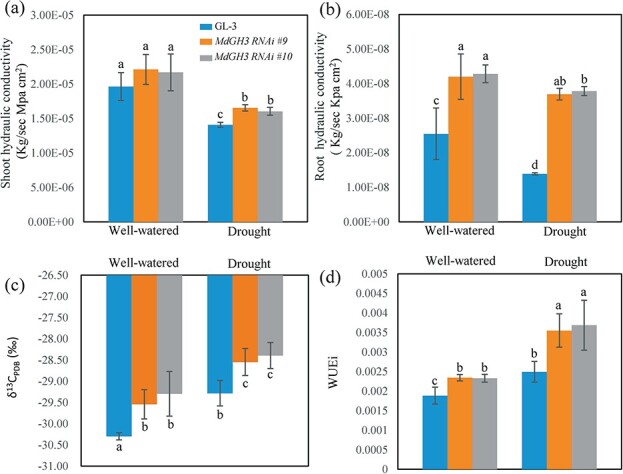
*MdGH3* RNAi plants had greater hydraulic conductivity and higher water use efficiency (WUE) after long-term drought stress. **a**, **b** Shoot hydraulic conductivity (**a**) and root hydraulic conductivity (**b**) of GL-3 and *MdGH3* RNAi plants under well-watered and long-term drought stress. **c** WUE was determined by carbon isotope composition (δ^13^C). Plants were exposed to drought for 60 days. **d** Intrinsic WUE. Error bars indicate the standard deviation (*n* = 7). One-way ANOVA (Tukey test) was performed.

Prolonged drought stress decreased both root and shoot hydraulic conductivity (Fig. 3a and b). However, drought stress affected both parameters more severely in GL-3 plants, and the effect on root hydraulic conductivity was particularly pronounced. Under drought stress, the root hydraulic conductivity of GL-3 plants decreased by 45% and that of *MdGH3* RNAi plants decreased by only 12% (Fig. 3b). Compared with GL-3 plants, *MdGH3* RNAi plants had significantly higher root and shoot hydraulic conductivity under drought stress (Fig. 3a and b).

WUE represents a fundamental physiological trade-off in plants, and higher WUE is favorable in consistently water-limited environments [21]. Using carbon isotope composition (δ^13^C) as a physiological indicator of WUE, we found that drought stress increased the WUE of all plants. However, *MdGH3* RNAi plants had a considerably higher WUE than GL-3 plants under both well-watered and drought conditions (Fig. 3c). In addition, intrinsic WUE (WUEi), which was measured as the ratio of photosynthesis rate/transpiration rate, was higher in *MdGH3* RNAi plants compared with GL-3 under drought stress (Fig. 3d).

We also evaluated long-term drought stress tolerance with the same plants for an additional year and obtained similar findings. Under long-term drought stress, *MdGH3* RNAi plants were taller, had higher photosynthetic rates, higher root and shoot hydraulic conductivity, and thicker stem diameters (Supplementary Data Fig. S3), suggesting that *MdGH3* RNAi plants grow better under prolonged drought stress.

### Grafting ‘Fuji’ onto *MdGH3* RNAi plants enhances drought tolerance

**Figure 4:**
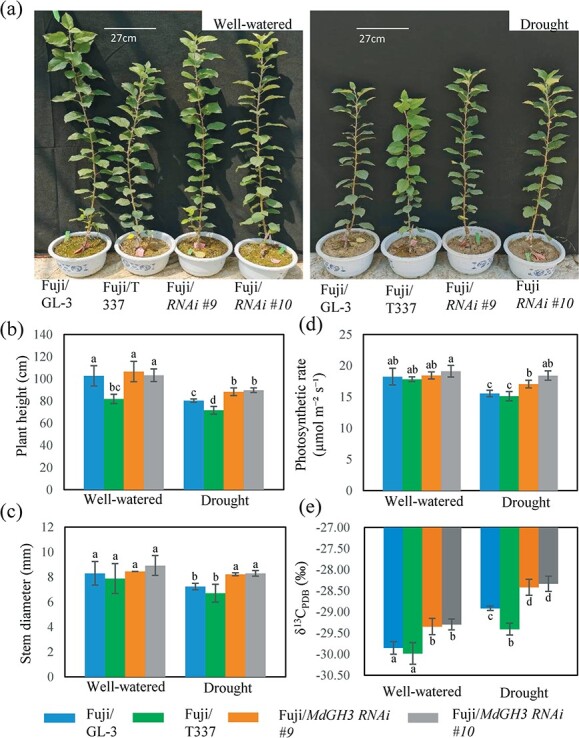
‘Fuji’ plants grafted onto the *MdGH3* RNAi rootstock were more tolerant to long-term drought stress. Plant morphology (**a**), plant height (**b**), stem diameter (**c**), photosynthetic rate (**d**), and water use efficiency (**e**) of ‘Fuji’ plants grafted on GL-3, M9-T337, and *MdGH3* RNAi plants under well-watered and long-term drought stress. Plants were exposed to drought for 60 days. Error bars indicate the standard deviation (*n* = 5–7). One-way ANOVA (Tukey test) was performed.

Stress-resistant rootstocks are critical for fruit tree cultivation. To examine the effect of *MdGH3* RNAi plants on scion drought tolerance, we grafted the ‘Fuji’ scion onto GL-3, *MdGH3* RNAi (RNAi), or M9-T337 rootstock. M9-T337 is a widely used dwarf apple rootstock. We then exposed the grafted plants to long-term drought stress. Under well-watered conditions, ‘Fuji’/M9-T337 plants were shorter, but there was no difference in height between ‘Fuji’/GL-3 and ‘Fuji’/RNAi plants. Under drought stress, ‘Fuji’/M9-T337 plants were the shortest, and ‘Fuji’/RNAi plants were significantly taller than ‘Fuji’/GL-3 plants (Fig. 4a and b). Stem diameter showed a pattern similar to plant height, although diameter did not differ among grafting combinations under well-watered conditions (Fig. 4c). Long-term drought stress decreased the photosynthetic rate and increased the WUE of all grafted plants. Under drought, the decrease in photosynthetic rate was lower in ‘Fuji’/RNAi plants (~7%) than in ‘Fuji’/GL-3 and ‘Fuji’/M9-T337 plants (~15%). Therefore, the photosynthetic capacity of ‘Fuji’/RNAi plants under drought was higher than that of ‘Fuji’/GL-3 and ‘Fuji’/M9-T337 plants, and the photosynthetic capacity of the latter two graft combinations did not significantly differ (Fig. 4d). ‘Fuji’/RNAi plants had consistently higher specific leaf weight and lower specific leaf area (Supplementary Data Fig. S4a and b). Moreover, stable carbon isotope composition analysis showed that ‘Fuji’/RNAi plants had higher WUE than ‘Fuji’/GL-3 and ‘Fuji’/M9-T337 plants under both well-watered and drought stress conditions. ‘Fuji’/M9-T337 plants had the lowest WUE under drought (Fig. 4e). Together, these findings indicate that using *MdGH3* RNAi plants as rootstocks can facilitate scion performance under long-term drought.

### Grafting ‘Fuji’ onto *MdGH3* RNAi plants promotes flowering and fruiting after drought

Flowering and fruit setting are of great concern for fruit trees. M9-T337 is a dwarf rootstock that can promote early flowering and early fruiting. To study whether drought-tolerant *MdGH3* RNAi plants affect the flowering and fruiting of scions when used as the rootstock, we observed the flowering and fruiting of ‘Fuji’ scions grafted onto different rootstocks 2 or 3 years after grafting.

**Figure 5:**
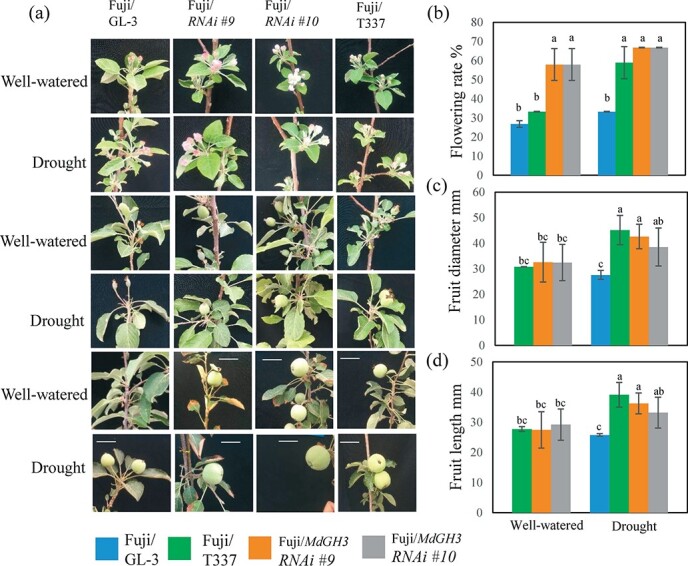
Grafting ‘Fuji’ onto *MdGH3* RNAi plants promotes flowering, fruit setting, and fruit size of the scion after drought. **a** Flowering and fruit setting of ‘Fuji’ grafted onto GL-3, M9-T337, and *MdGH3* RNAi plants after 3 years. Scale bars = 5 cm. Well-watered, all plants were regularly watered. Drought, all plants were subjected to long-term drought stress for 60 days in the first year and then watered regularly. **b**–**d** Flowering rate (**b**), fruit diameter (**c**), and fruit length (**d**) of the grafted plants shown in (**a**). Error bars indicate the standard deviation (*n* = 5). One-way ANOVA (Tukey test) was performed.

In the well-watered group, only ‘Fuji’/M9-T337 plants had buds and fruits in the second year (Supplementary Data Fig. S6). Three years after grafting, ‘Fuji’/RNAi plant buds were larger and bloomed earlier than ‘Fuji’/GL-3 plants (Fig. 5a). In addition, the flowering rate of ‘Fuji’/RNAi plants was higher than that of ‘Fuji’/M9-T337 and ‘Fuji’/GL-3 plants (Fig. 5b). Notably, ‘Fuji’/GL-3 plants did not bear fruit, though they bloomed in the spring (Fig. 5a). There was no difference in fruit size between ‘Fuji’/RNAi and ‘Fuji’/M9-T337 plants (Fig. 5c and d).

After treatment with prolonged drought stress for 2 months, the grafted plants were watered normally. In the second year after grafting, ‘Fuji’/M9-T337 plants and ‘Fuji’/RNAi plants bloomed and bore fruits, except for ‘Fuji’/GL-3 plants (Supplementary Data Fig. S6). Three years after grafting, the ‘Fuji’/RNAi plant buds were larger and bloomed earlier than the ‘Fuji’/GL-3 plants. The flowering rate of ‘Fuji’/RNAi plants was greater than that of ‘Fuji’/GL-3 plants (Fig. 5b). In addition, the fruits of ‘Fuji’/RNAi plants were set as early as those of ‘Fuji’/M9-T337 plants (Fig. 5a). Moreover, the fruit size of ‘Fuji’/RNAi plants was as large as that of ‘Fuji’/M9-T337 but larger than that of ‘Fuji’/GL-3 plants (Fig. 5c and d). Interestingly, we found that after long-term drought stress the fruits of all grafting combinations were larger than those under well-watered conditions (Fig. 5c and d). Overall, these findings indicate that the use of *MdGH3* RNAi plants as rootstocks can achieve the same effect as the M9-T337 rootstock to promote the early flowering, fruiting, and fruit size of the scion.

### Grafting ‘Fuji’ onto *MdGH3* RNAi plants increases C/N ratio after drought

**Figure 6:**
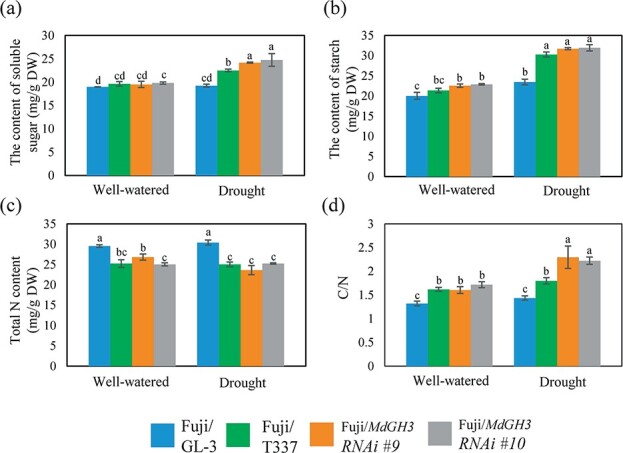
Grafting ‘Fuji’ onto the *MdGH3* RNAi plants facilitates the C/N ratio in the scion. Contents of soluble sugar (**a**), starch (**b**), and total N (**c**) and C/N ratio (**d**) in ‘Fuji’ grafted onto GL-3, *MdGH3* RNAi, and M9-T337 plants after 3 years. Well-watered, all plants were regularly watered. Drought, all plants were subjected to long-term drought stress for 60 days in the first year and then watered regularly. Error bars indicate the standard deviation (*n* = 3). One-way ANOVA (Tukey test) was performed.

To investigate the mechanisms of early flowering, we measured the contents of soluble sugar, starch, and total N in scions of different grafting combinations. Among the drought-treated grafting combinations, we found that the contents of soluble sugar and starch of ‘Fuji’/RNAi plants and ‘Fuji’/M9-T337 plants were higher than those of ‘Fuji’/GL-3 plants (Fig. 6a and b), while the total nitrogen content of ‘Fuji’/RNAi plants and ‘Fuji’/M9-T337 plants was lower than that of ‘Fuji’/GL-3 plants (Fig. 6c). Furthermore, the C/N ratio of ‘Fuji’/RNAi plants was higher than that of ‘Fuji’/GL-3 plants and similar to that of ‘Fuji’/M9-T337 plants (Fig. 6d). These results indicate that the high C/N ratio might contribute to the early flowering of ‘Fuji’/RNAi plants.

### Grafting ‘Fuji’ onto *MdGH3* RNAi plants promotes fruit size and fruit quality after drought

**Figure 7:**
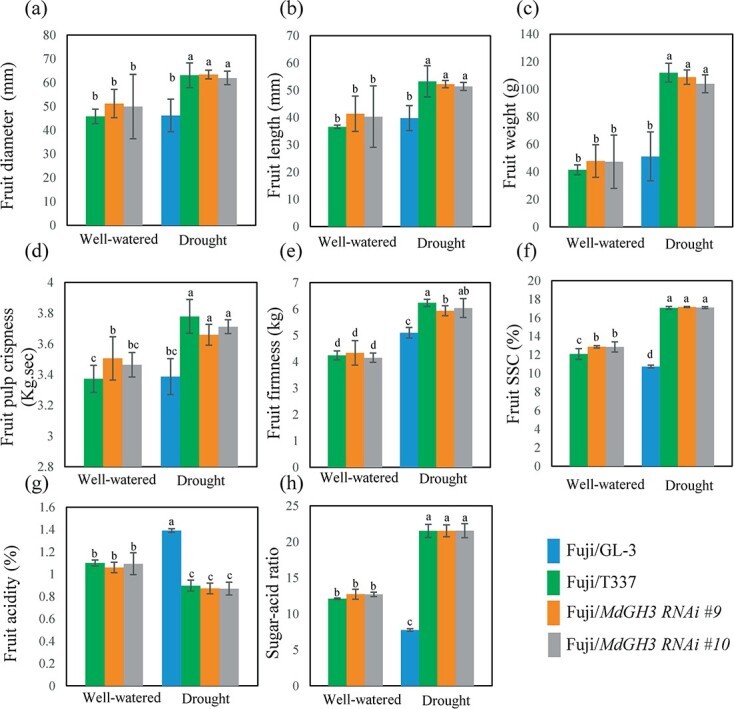
Grafting ‘Fuji’ onto *MdGH3* RNAi plants promotes fruit size and quality of the scion after drought. Fruit diameter (**a**), fruit length (**b**), fruit weight (**c**), fruit pulp crispness (**d**), fruit firmness (**e**), fruit soluble solids content (SSC, **f**), fruit acidity (**g**), and sugar/acid ratio (**h**). Well-watered, all plants were regularly watered. Drought, all plants were subjected to long-term drought stress for 60 days in the first year and then watered regularly. Error bars indicate the standard deviation (*n* = 5). One-way ANOVA (Tukey test) was performed.

Fruit yield and quality are two crucial parameters for the industry. Therefore, we next measured fruit weight and quality at the ripening stage. In the well-watered group, there was no difference in fruit weight and fruit quality between ‘Fuji’/RNAi and ‘Fuji’/M9-T337 plants (Fig. 7; Supplementary Data Figs S8 and S9).

Among the drought-treated grafting combinations, ‘Fuji’/RNAi and ‘Fuji’/M9-T337 plants had similar fruit size parameters (fruit diameter, fruit length, and fruit weight) and fruit quality parameters (crispness, firmness, SSC, acidity, sugar/acid ratio, peel hardiness, brightness, red degree, and yellow degree), except for fruit peel ductility (Fig. 7; Supplementary Data Figs S8 and S9b). Except for fruit red degree and yellow degree, which showed no difference among all grafting combinations, the fruits of ‘Fuji’/RNAi and ‘Fuji’/M9-T337 plants had higher fruit weight, stronger fruit brightness, higher fruit peel hardness, better fruit peel ductility, greater fruit pulp crispness, improved fruit firmness, improved fruit soluble solid content, lower fruit acidity, and improved sugar/acid ratio, compared with the fruits of ‘Fuji’/GL-3 plants (Fig. 7; Supplementary Data Figs S8, and S9). These results suggest that the use of *MdGH3* RNAi plants as rootstocks can achieve an effect similar to that of the M9-T337 rootstock without sacrificing fruit size and fruit quality of the scion.

### Expression of fruit size-related genes in scion fruits

To understand the molecular basis of fruit enlargement of ‘Fuji’/RNAi plants, we measured the expression levels of five genes associated with fruit size in the fruits of the scions: *Mdm-miR172p*, *MdAP2*, *MdARF6*, *MdEXP1*, and *MdEXP10* (*EXP*, expansion). Previous studies have shown that miRNA172p inhibits apple fruit growth by negatively regulating APETALA2 (AP2) [57]. AP2 is required for sepal development [58], which contributes to apple fruit size [57]. ARF6 is upregulated in the later stages of apple fruit development [59]. In addition, cell expansion can also affect fruit size [60–62]. The expression of *MdAP2* and *MdARF6* was upregulated in the fruits of ‘Fuji’/RNAi and ‘Fuji’/M9-T337 under drought. The expression of *MdEXP1* and *MdEXP10*, homologs of *EXP1*and *EXP10*, was also upregulated in the fruits of ‘Fuji’/RNAi and ‘Fuji’/M9-T337 under drought stress, while the expression of *Mdm-miR172p* was downregulated in the fruits of ‘Fuji’/RNAi and ‘Fuji’/M9-T337 (Supplementary Data Fig. S10). These results suggest that altered expression of these genes may contribute to the enlarged fruit of ‘Fuji’/RNAi.

## Discussion

We engineered transgenic apple plants with reduced expression of six *MdGH3* genes. The transgenic *MdGH3* RNAi plants displayed increased tolerance to long-term drought stress with greater growth, photosynthetic capacity, root system biomass, root and shoot hydraulic conductivity, root-to-shoot ratio, and WUE. When the ‘Fuji’ scion was grafted onto *MdGH3* RNAi plants, their drought stress tolerance and flowering rate improved when compared with ‘Fuji’ grafted onto GL-3 and M9-T337 rootstocks. In addition, using *MdGH3* RNAi plants as rootstocks did not reduce the fruit weight and quality of the scion compared with M9-T337 rootstocks. We also verified that the *NPTII* genes (kanamycin resistance determinants) could not be moved from the *MdGH3* RNAi rootstock to the ‘Fuji’ scion (Supplementary Data Fig. S5), confirming that there was no transgene risk to humans or the environment. The use of *MdGH3* RNAi plants as rootstocks to improve scion performance under drought stress is therefore feasible.

Root system architecture plays a vital role in the drought tolerance of plants, including fruit trees [18, 63, 64]. Generally, root system size is positively correlated with drought tolerance. A higher root-to-shoot ratio directly reflects the drought tolerance of plants under drought conditions. Increases in the root-to-shoot ratio improve the survival rate of plants under drought stress [65]. In our study, we found that the roots of *MdGH3* RNAi transgenic plants were stronger than those of GL-3 after long-term drought treatment (Fig. 2b and c), and *MdGH3* RNAi plants had a higher root-to-shoot ratio compared with GL-3 plants (Fig. 2d). The stronger root systems of *MdGH3* RNAi transgenic plants may be related to the function of the *GH3* genes themselves. Previous research has shown that *GH3* genes participate in auxin homeostasis by conjugating excess IAA to amino acids [43, 44, 66]. Overexpression of *OsGH3-2* in rice decreases IAA levels, resulting in fewer crown roots and root hairs [52]. Overexpression of *MsGH3.5* in the apple rootstock M26 observably reduces the IAA levels but increases the cytokinin levels, resulting in fewer adventitious roots [66]. In our study, we found that *MdGH3* RNAi plants had higher IAA content than GL-3 in leaves and roots after long-term drought stress(Supplementary Data Figs S1b and S2). Therefore, the stronger root systems of *MdGH3* RNAi plants under long-term drought stress may be attributed to their increased IAA content.

WUE is positively correlated with drought tolerance of plants [7, 20, 21], including apple [7, 20, 31], and is an important index of plant adaptation to drought stress [21]. WUE can vary with the photosynthetic rate, stomatal conductance, or both [21]. *MhYTP1* overexpression improves drought tolerance and WUE in apple plants by increasing ABA levels and stomatal density, and reducing the stomatal aperture [31]. Previous studies have also shown that changes in root structure can improve the capacity of plants to absorb water as well as WUE under drought stress [15–17, 67]. Rice varieties with greater root weight and longer root length have higher WUE [67]. In our study, *MdGH3* RNAi plants had higher photosynthetic rates, stronger root systems, and higher root hydraulic conductivity than GL-3 plants under prolonged drought stress (Figs 2 and 3), indicating that the higher WUE in *MdGH3* RNAi plants might be attributed to their high photosynthesis rate and strong root system.

Grafting is a viable method for improving scion growth, early flowering, fruit quality, and stress resistance [27, 29, 31, 37, 38, 68]. The use of salt-tolerant rootstock improves the yield of pepper (the scion) under salinity stress by maintaining photosynthetic capacity and sink strength [29]. In addition, grafting can improve drought tolerance of scions by modifying antioxidant enzyme activities [38], improving photosynthetic capacity, and decreasing reactive oxygen species accumulation under drought stress in the scions [68].

We found that using *MdGH3* RNAi plants as rootstocks improves scion performance under drought conditions. Firstly, using *MdGH3* RNAi plants as rootstocks may have a positive effect on growth. *MdGH3* RNAi plants developed a stronger root system and were more effective in absorbing and transporting water and nutrients to the shoot compared with GL-3 plants. ‘Fuji’/RNAi plants not only had greater diameters but were also taller and had higher photosynthetic capacity, WUE, and specific leaf dry weight compared with ‘Fuji’/GL-3 plants (Fig. 4; Supplementary Data Fig. S4). The strong roots of *MdGH3* RNAi plants might play a key role in improving the drought tolerance of scions. Previous studies have demonstrated communication of the genetic material between the stock and the scion [69, 70]. Grafting to *msh1* rootstock leads to heritable and enhanced growth vigor of scion progeny in *Arabidopsis* and tomato, which is associated with the small interfering RNA (siRNA)-directed DNA methylation (RdDM) process [69]. Communication between transgenic *MdGH3* RNAi plants and scions should also occur; however, additional studies are required to confirm this. Secondly, the use of *MdGH3* RNAi plants as rootstocks can achieve the same effect as the M9-T337 rootstock in promoting early flowering, fruit setting, fruit weight, and fruit quality of the scions after drought (Figs 5 and 7). A high C/N ratio has been found to promote the early flowering of plants [42]. We measured the C/N ratio in scions of different grafting combinations and found that the C/N ratio of ‘Fuji’/RNAi plants was higher than that of ‘Fuji’/GL-3 plants and was similar to that of ‘Fuji’/M9-T337 plants (Fig. 6d). Among the drought-treated grafting combinations, we found that the fruit size of ‘Fuji’/RNAi plants was similar to that of ‘Fuji’/M9-T337 but larger than that of ‘Fuji’/GL-3 plants (Fig. 7a–c). Our previous research demonstrated that *MdGH3* RNAi plants contain more IAA under well-watered and short-term drought conditions [55]. In the current study, we also measured the IAA content in the leaves and roots of *MdGH3* RNAi plants and GL-3 plants. We found that *MdGH3* RNAi plants had more IAA under long-term droughtconditions (Supplementary Data Figs S1b and S2). However, to our surprise there was no significant difference in IAA content among all grafting combinations (Supplementary Data Fig. S7), implying that IAA might not contribute to the larger fruit size of ‘Fuji’/RNAi and ‘Fuji’/M9-T337. Therefore, it is possible that transgenic rootstocks may affect scion fruit size through cell expansion and the miRNA172-AP2 pathway.

In sum, we engineered transgenic apple plants with reduced expression of six *MdGH3* genes. The *MdGH3* RNAi plants displayed improved drought tolerance, WUE, hydraulic conductivity, root-to-shoot ratio, and photosynthetic capacity under drought stress. We found that they could be used as rootstocks to improve scion performance under drought conditions without penalty to fruit yield and quality. These results define the functions of six *MdGH3* genes in promoting tolerance to drought conditions and suggest that these six *MdGH3* genes can serve as candidate genes for the breeding of apple drought tolerance using biotechnology.

## Materials and methods

### Plant materials, growth conditions, and stress treatment

Long-term drought treatment was described previously [18, 55]. Briefly, 36 seedlings of non-transgenic plants (GL-3, selected from progenies of *M.* × *domestica* ‘Royal Gala’) or *MdGH3* RNAi plants [55] (knockdown of *MdGH3.6* and five close paralogs from *Malus* × *domestica*) were transferred to pots (30 cm × 18 cm) filled with equal parts of local loess sand and wormcast medium. Pots were placed in a greenhouse under natural illumination, with a humidity of 35–55% and a temperature of 20–35°C for 1 month. Fourteen grafted plants were grown in the same environment. Plants of each line were divided into two groups: a well-watered group (field capacity of 75–85%, *n* = 18 or 7) and a long-term drought group (field capacity of 45–55%, *n* = 18 or 7). Both treatments lasted ~60 days.

Plant height was measured with a meter ruler. Stem diameter, fruit diameter, and fruit length were measured with a Vernier caliper. Photosynthetic rate, intercellular CO_2_ concentration, stomatal conductance, and transpiration rate were monitored with an LI-6400XT (LI-COR, USA). Leaf area was recorded with a scanner. Leaf weight and dry weight of roots and stems were measured with an electronic balance. Data were collected from the fifth to ninth leaves from the base of the stems.

### Measurement of sugar, starch, and total N content

About 0.5 mg of the dry weight of leaves was used for soluble sugar and starch extractions, and the contents were determined by an anthrone colorimetric method [71]. Approximately 0.5 mg of dry weight of leaves was used for total N extraction, and the content was determined as described previously [72]. The fifth to ninth leaves from the base of the stems were used as plant materials.

### Measurement of hydraulic conductivity

Hydraulic conductivity of roots and shoots was determined with a high-pressure flow meter (HPFM, Dynamax, Houston) according to previous methods [18, 73, 74].

### Stable carbon isotope analysis

Stable carbon isotope analysis (δ^13^C) was performed according to previous descriptions [20] in the Third Institute of Oceanography, Ministry of Natural Resources in China.

### Measurement of fruit weight and quality

Fruit weight was measured with an electronic scale. Fruit diameter and fruit length were measured with a Vernier caliper. The color indicators (brightness, red degree, and yellow degree) were recorded with a chromatic meter. Fruit firmness was measured with a hardness tester. Fruit pulp crispness, fruit peel hardness, and fruit peel ductility were monitored with a texture analyzer. Fruit soluble solids content was measured by a saccharimeter, and fruit acidity was measured by an acidimeter. Data were collected from five fruits. One fruit was used as one biological replicate.

### RNA isolation and RT–qPCR analysis

About 0.5 g of scion fruit pulp from all grafting combinations under well-watered and drought conditions was used for RNA extraction as described previously [55]. RT–qPCR analysis was performed as described previously [55]. The primers are shown in Supplementary Data Table S1.

### Indole-3-acetic acid measurements

The IAA content of leaves from all scions was extracted and quantified according to the methods described by Jiang *et al*. [55]. In brief, 1 g of frozen powder of leaf sample was extracted for 16 hours with hormone extraction buffer at 4°C in the dark. The content of IAA was quantitatively analyzed by HPLC–MS/MS (QTRAP 5500).

### Statistical analysis

Data are reported as the mean ± standard deviation. Statistical significance was determined by one-way ANOVA (Tukey’s test) using SPSS (version 21.0). Variations were considered significant if *P* < .05, .01, or .001.

## Acknowledgements

This research was supported by the National Key Research and Development Program of China (2019YFD1000100) and the National Natural Science Foundation of China (31872080).

## Author contributions

Q.G. designed the project; L.J., W.S., and C.L. conducted experiments and data analysis; L.J .and Q.G. wrote the manuscript; M.M.T., X.L., F.M., and S.Z. helped with the discussion of the work. All the authors were involved in the preparation of the final manuscript.

## Data availability

Sequence information used in this article can be found in the Genome Database for Rosaceae with the following gene numbers: MDP0000281079 (*Mdm-miRNA172p*), MDP0000137561 (*MdAP2)*, MDP0000268306 (*MdARF6*), MDP0000681724 (*MdEXP1*), and MDP0000560112 (*MdEXP10*).

## Conflict of interest

The authors declare that they have no conflicts of interest.

## Supplementary data

Supplementary data is available at *Horticulture Research* online.

## Supplementary Material

Web_Material_uhac122
