# Supplementary material for: Engineering drought-tolerant apple by knocking down six GH3 genes and potential application of transgenic apple as a rootstock
Source: Hortic Res. 2022 May 26;9:uhac122. doi: 10.1093/hr/uhac122 (PMC9347023; doi:10.1093/hr/uhac122)
Supplement: Web_Material_uhac122 [file web_material_uhac122.zip › GH3 RNAi as rootstock paper-sup figures-20220424.pdf]

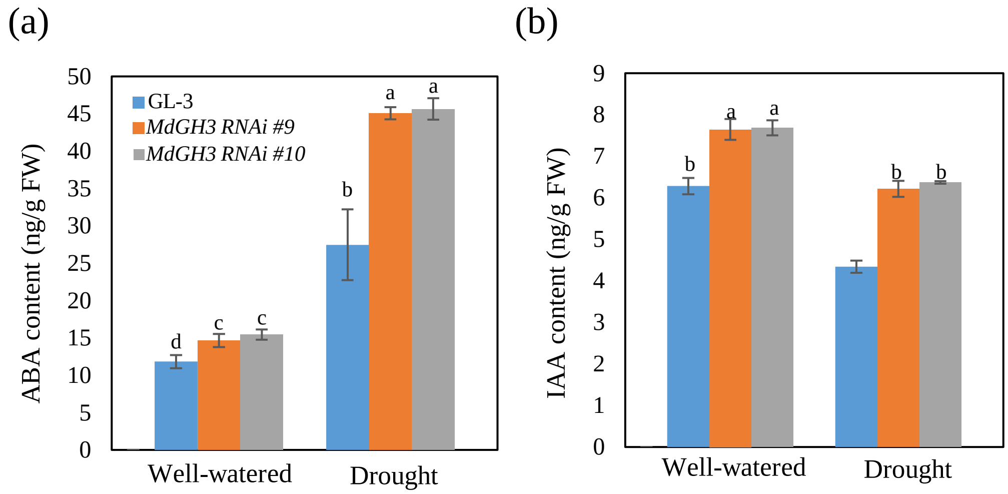

Fig. S1 The ABA and IAA content of leaves in GL-3 and *MdGH3* RNAi plants under well-watered and long-term drought stress. Plants were exposed to drought for 60 d. Error bars indicate SD (n = 3). One-way ANOVA (Tukey test) was performed.

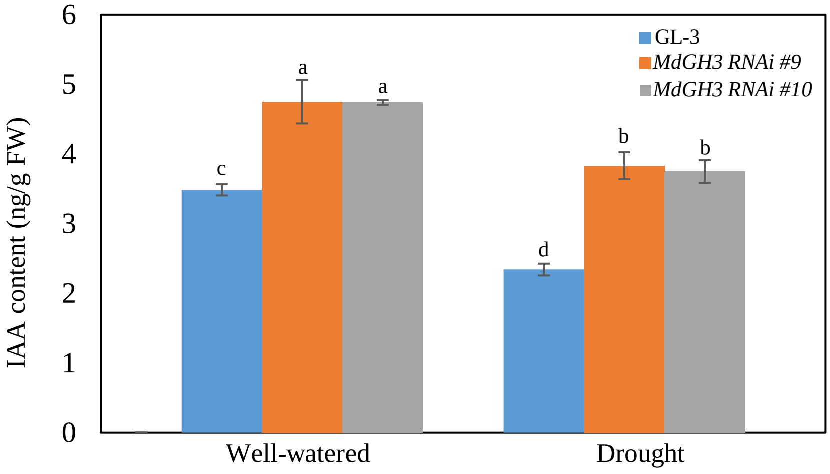

Fig. S2 The IAA content of roots in GL-3 and *MdGH3* RNAi plants under well-watered and long-term drought stress. Plants were exposed to drought for 60 d. Error bars indicate SD (n = 3). One-way ANOVA (Tukey test) was performed.

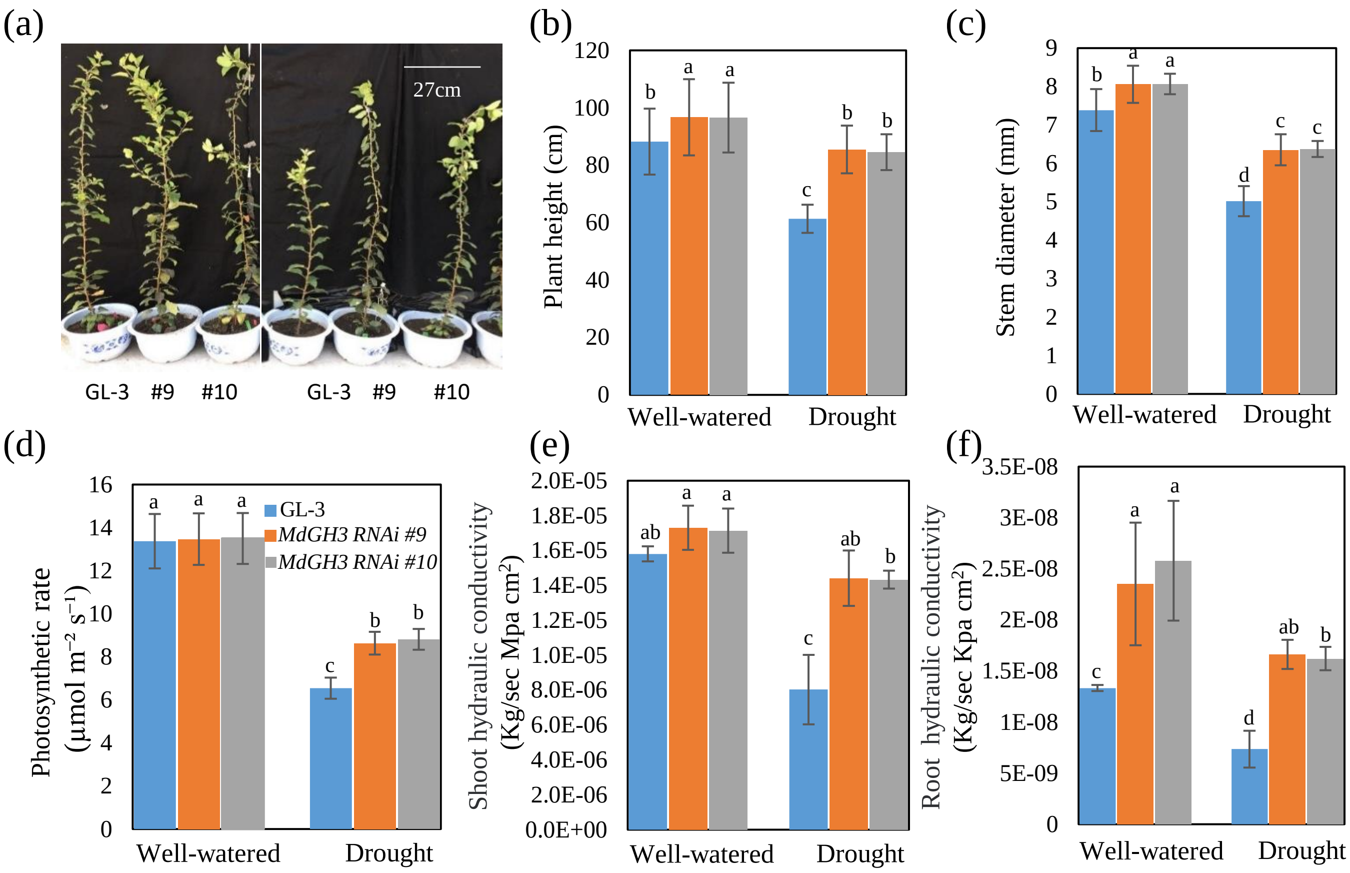

Fig. S3 *MdGH3* RNAi plants were more tolerant to long-term drought stress. (a)- (f) Plant morphology (a), height (b), stem diameter (c), photosynthetic rate (d), shoot hydraulic conductivity (e), and root hydraulic conductivity (f) of GL-3 and *MdGH3* RNAi plants under well-watered and long-term drought stress. Plants were exposed to drought for 60 d. Error bars indicate SD (n = 18). One-way ANOVA (Tukey test) was performed.

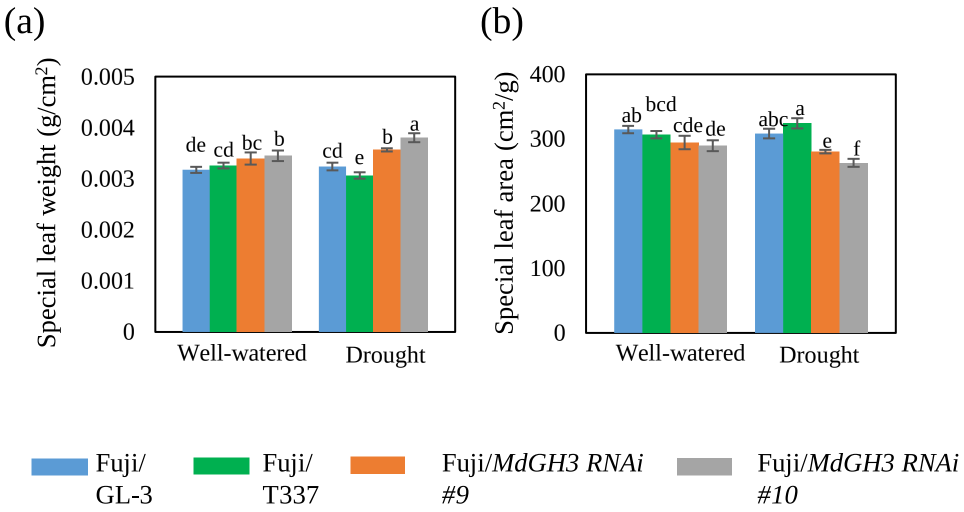

Fig. S4 Special leaf weight and special leaf area of Fuji (the scion) grated onto rootstocks including GL-3, M9-T337, or *MdGH3* RNAi plants under well-watered and drought conditions. (a) Special leaf weight. (b) Special leaf area. Plants were treated with drought for 60 d and leaves were collected for the measurements. Error bars indicate SD (n = 7). One-way ANOVA (Tukey test) was performed.

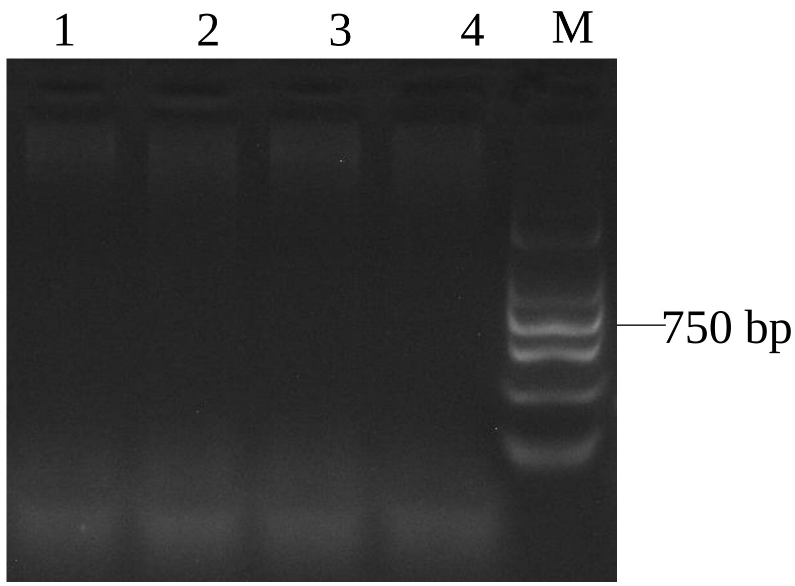

Fig. S5 Detection of NPTII (Kanamycin resistance gene) in the scion of Fuji grafted on *MdGH3* RNAi rootstock. Four-month-old grafted plants were used. Lane 1, Fuji scion grafted onto GL-3. Lane 2, Fuji scion grafted onto M9-T337. Lane 3, Fuji scion grafted onto *MdGH3* RNAi plants (#9). Lane 4, Fuji scion grafted onto *MdGH3* RNAi plants (#10). M, DNA Marker.

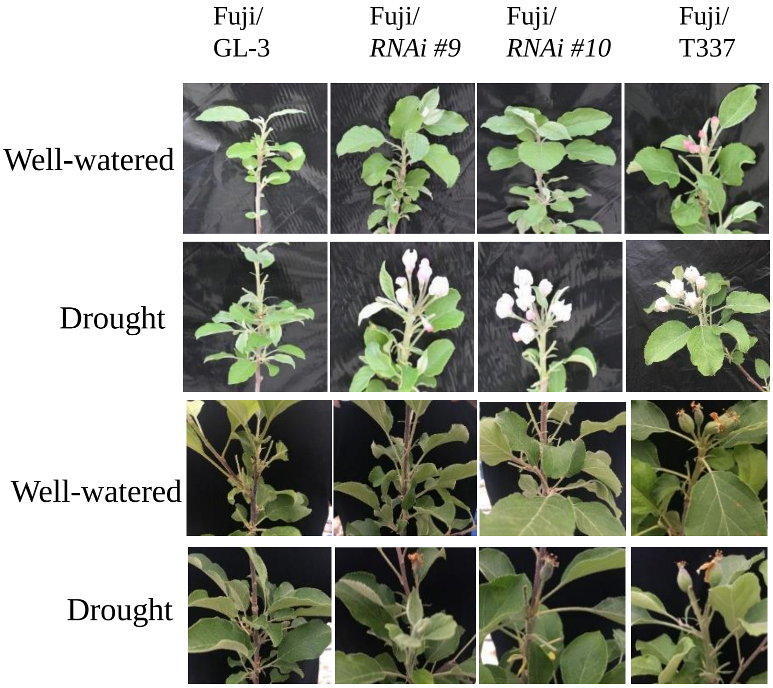

Fig. S6 Flowering and fruit setting of Fuji grafted onto GL-3, M9-T337, *MdGH3* RNAi plants after two years. Well-watered, all plants were regularly watered. Drought, all plants were subjected to long-term drought stress for 60 d in the first year, and then watered regularly.

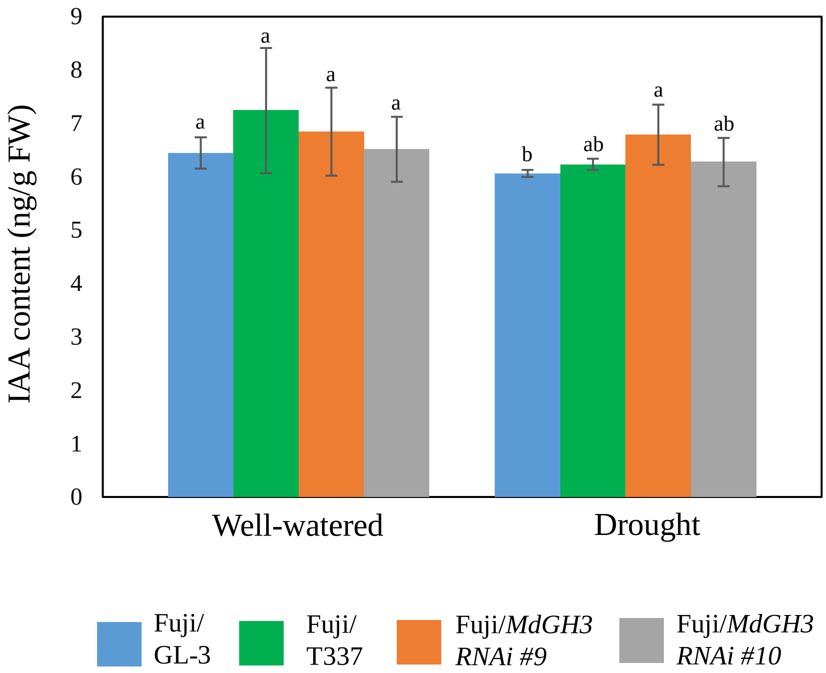

Fig. S7 The IAA content of leaves in scions. IAA content of leaves in Fuji grafted onto GL-3, *MdGH3* RNAi plants, or M9-T337 plants for three years. Well-watered, all plants were regularly watered. Drought, all plants were subjected to long-term drought stress for 60 d in the first year, and then watered regularly. Error bars indicate SD (n = 3). One-way ANOVA (Tukey test) was performed.

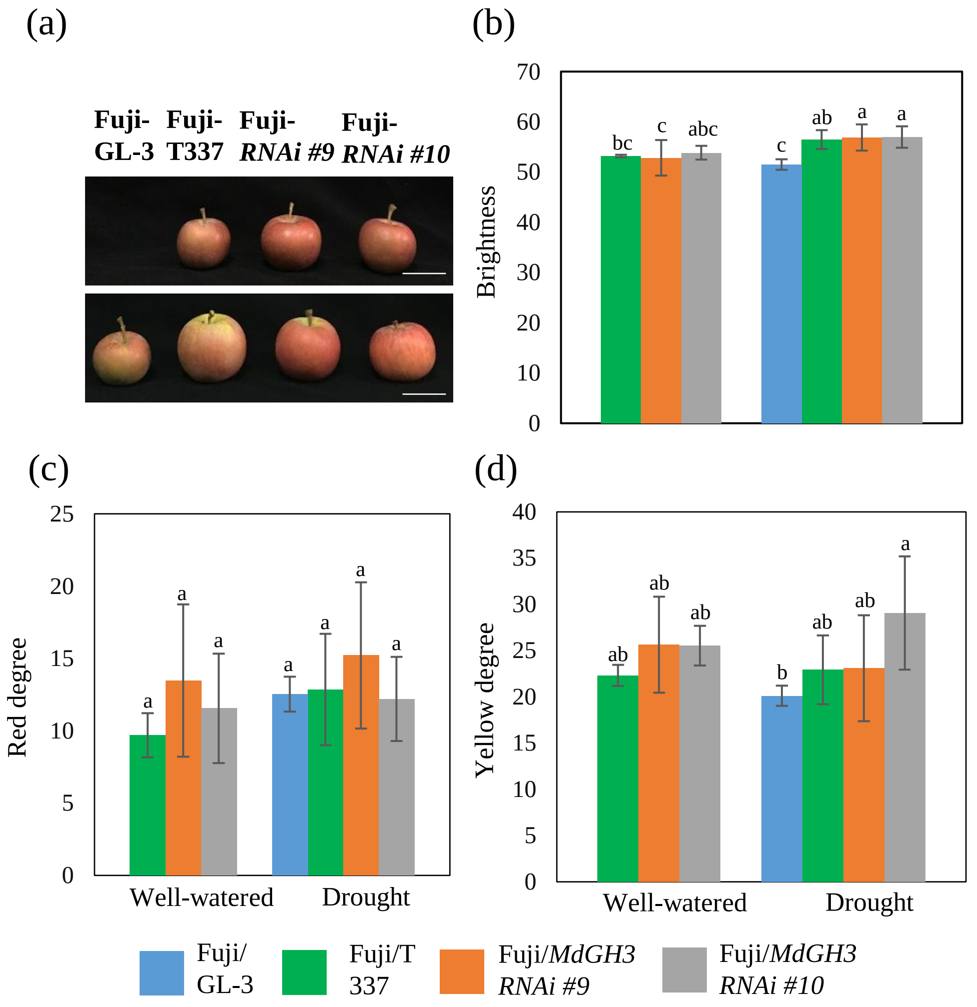

Fig. S8 The external quality of apple fruits in all the scions under well-watered and drought. (a)- (d) Fruit appearance (a), brightness (b), red degree (c), and yellow degree (d). Bars = 4 cm. Well-watered, all plants were regularly watered. Drought, all plants were subjected to long-term drought stress for 60 d in the first year, and then watered regularly. Error bars indicate SD (n = 5). One-way ANOVA (Tukey test) was performed.

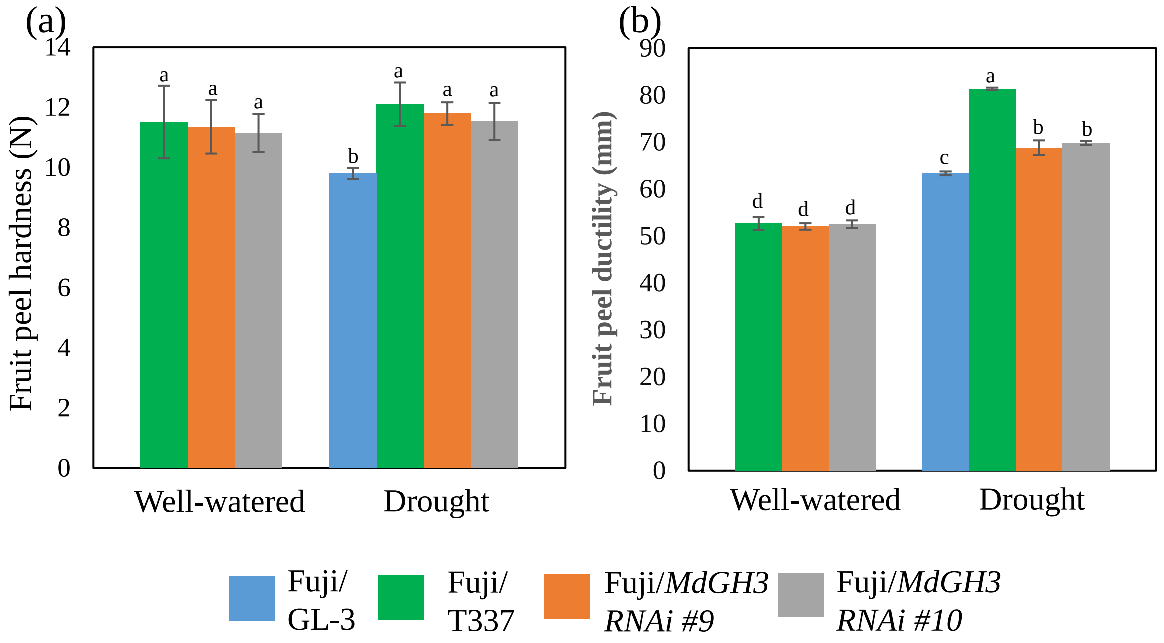

Fig. S9 The fruit peel characteristics of apple fruits in all the scions under well-watered and drought. (a) Fruit peel hardness, (b) Fruit peel ductility. Well-watered, all plants were regularly watered. Drought, all plants were subjected to long-term drought stress for 60 d in the first year, and then watered regularly. Error bars indicate SD (n = 5). One-way ANOVA (Tukey test) was performed.

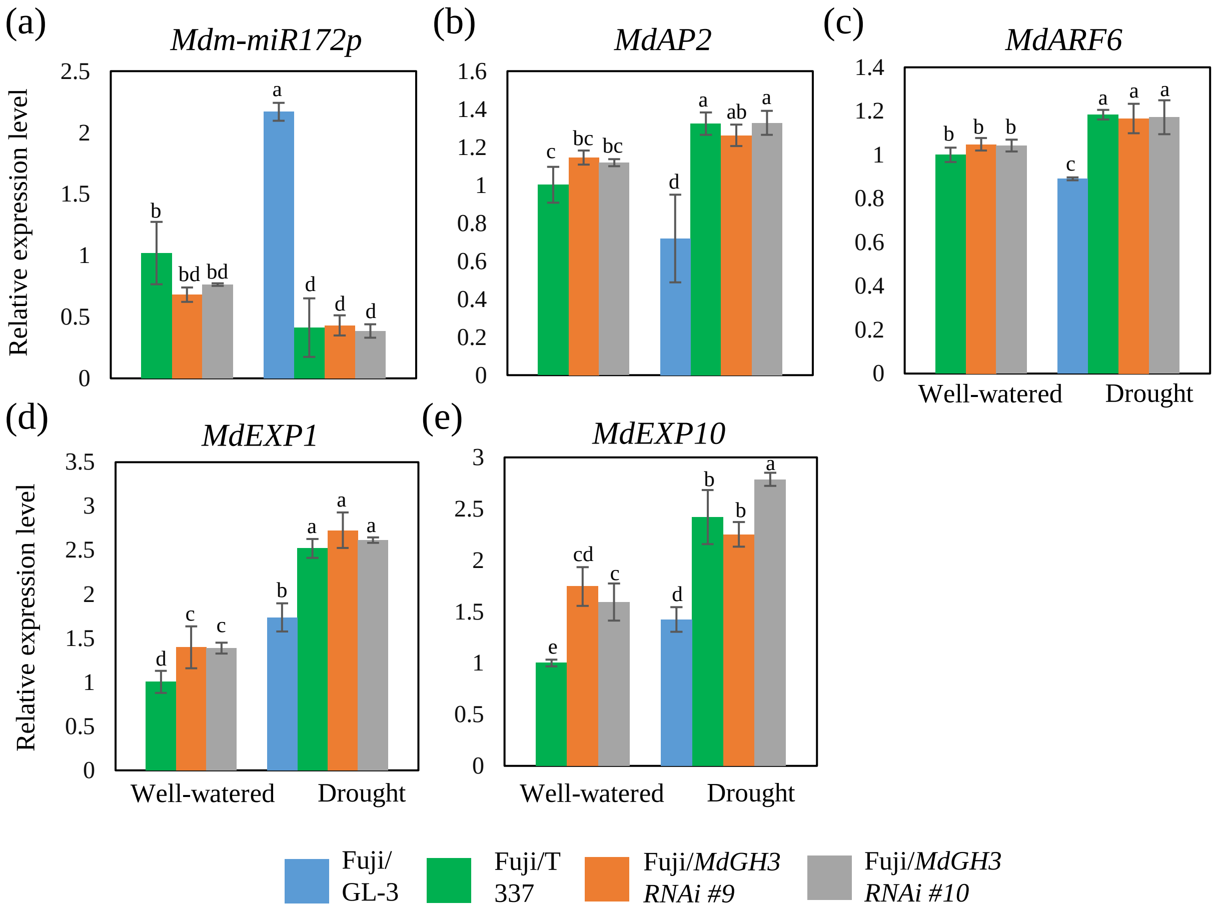

Fig. S10 Expression levels of genes related to fruit size in the fruits of the scions under well-watered and drought. Well-watered, all plants were regularly watered. Drought, all plants were subjected to long-term drought stress for 60 d in the first year, and then watered regularly. Error bars indicate SD (n = 3). One-way ANOVA (Tukey test) was performed.
